# The Political Economy of the Mental Health System: A Marxist Analysis

**DOI:** 10.3389/fsoc.2021.771875

**Published:** 2022-01-17

**Authors:** Joanna Moncrieff

**Affiliations:** Division of Psychiatry, University College London, London, United Kingdom

**Keywords:** Marxism, mental disorder, history of psychiatry, neoliberalism, political economy

## Abstract

The present paper analyses the functions of the mental health system in relation to the economic organisation of society, using concepts derived from Marx’s work on political economy and building on previous critiques. The analysis starts from the position that mental health problems are not equivalent to physical, medical conditions and are more fruitfully viewed as problems of communities or societies. Using the example of the United Kingdom, it traces how a public mental health system evolved alongside capitalism in order to manage the problems posed by people whose behaviour was too chaotic, disruptive or inefficient to participate in a labour market based on exploitation. The system provided a mixture of care and control, and under recent, Neoliberal regimes, these functions have been increasingly transferred to the private sector and provided in a capitalistic manner. Welfare payments are also part of the system and support those less seriously affected but unable to work productively enough to generate surplus value and profit. The increased intensity and precarity of work under Neoliberalism has driven up benefit claims at the same time as the Neoliberal state is trying to reduce them. These social responses are legitimised by the idea that mental disorders are medical conditions, and this idea also has a hegemonic function by construing the adverse consequences of social and economic structures as individual problems, an approach that has been particularly important during the rise of Neoliberalism. The concept of mental illness has a strategic role in modern societies, therefore, enabling certain contentious social activities by obscuring their political nature, and diverting attention from the failings of the underlying economic system. The analysis suggests the medical view is driven by political imperatives rather than science and reveals the need for a system that is more transparent and democratic. While the mental health system has some consistent functions across all modern societies, this account highlights one of the endemic contradictions of the capitalist system in the way that it marginalises large groups of people by narrowing the opportunities to make an economic contribution to society.

## Introduction

The subject of mental health has perhaps never been more widely discussed than today, and mental health problems more widely accepted as “proper” medical conditions. There has been a huge escalation in the diagnosis and treatment of such problems across western societies in the past few decades. A quarter of the English population report that they have suffered from a mental illness at some point in their lives (Health and Social Care Information Centre, 2015), and even larger numbers have been persuaded that many instances of unhappiness and discontent arise from biochemical abnormalities and require medical interventions (Pilkington et al., 2013). This phenomenon has been referred to as “psychiatrization” (Beeker et al., 2021), and also as widening medicalisation or “disease-mongering”, since psychiatric disorders are classified as a subset of medical disorders and often subject to medical-style interventions like pharmaceuticals (Conrad and Potter, 2000; Moynihan et al., 2002). In the meantime, there has been a profound reorganisation of provision for the seriously mentally unwell, with care provided by large state institutions transferred to smaller facilities and organisations, many run by the private sector on a “for-profit” basis.

The works of Marx and Engels are recognised to provide important insights into the nature and workings of many contemporary institutions, and systems for addressing mental health problems, particularly psychiatry, are no exception. Several scholars within a broadly defined Marxist tradition have examined mental institutions and treatments, building on the analysis of social deviance, and focusing on the way psychiatric interventions serve as mechanisms of social control, developed to manage behaviour that threatens to destabilise the capitalist system (Conrad, 1992; Scull, 1993; Cohen, 2016). Other authors have documented how, over recent decades, Neoliberal capitalism has coincided with the trend to medicalise and “commodify” more and more aspects of human feelings and behaviour, in the process turning them into a source of profit for the pharmaceutical and healthcare industries (Fisher, 2009; Davies, 2017). The ideological consequences of reframing social problems as individual pathology have also been highlighted, in the way this process diverts attention from the structural inequality and injustice that make life difficult for people in the first place (Fisher, 2009; Davies, 2011; Cohen, 2016).

Marxist analyses overlap with the “antipsychiatry” position, which argues that mental illness is a strategic, political concept, rather than a scientific one (Szasz, 1970; Szasz, 1989). There is also a wealth of Marxist literature on the welfare state that is relevant to understanding the role and functions of the mental health system (Gough, 1979; Higgs, 1993).

In the following article, I set out an analysis of how the mental health system relates to the economy, particularly a capitalist economy, making use of Marxist concepts such as use value, exchange value, exploitation, productive labour and ideology (see Table 1). I trace the evolution of the English system, revealing its social functions, which include social control, but also functions that have received little previous attention, such as the provision of care, and the way in which the biomedical ideology of psychiatry facilitates the capitalist welfare system, and promotes capitalist hegemony. I attempt to distinguish those aspects of the system that are specific to capitalism from those that are more general features of modern societies, and describe how understanding the mental health system in this way reveals some of the contradictions of capitalism. Since industrial capitalism is generally acknowledged to have started in England, the analysis provides a paradigmatic case of the relationship between economic development and social responses to mental disturbance in advanced capitalist economies, but it is not necessarily applicable to parts of the world where economic development has taken a different course.

**TABLE 1:** Marxist concepts.

Concept	
Use value	The value of a product in terms of the use it can be put to
Exchange value	The value of a product in terms of the money or other goods it can be exchanged for
Surplus value	The additional exchange value generated by labour over and above its own cost
Productive labour	Labour that generates surplus value
Exploitation	The accumulation of wealth through paying workers less than the value generated by their labour
Base/superstructure	The economic (or productive) system/social, political and cultural institutions and activities
Ideology	Ideas that support dominant class interests by obscuring the nature of reality

As a practising psychiatrist, I have experienced the situations that mental health services are required to address, and the frequent disjunction between the official diagnostic framework for explaining these situations, and the problems individuals, families and communities actually experience. Yet, I have also been socialised by the system, in particular by the language it employs. The terminology of “mental health,” “mental illness” and “mental disorder” is premised on the existence of a material entity or disease, located in the individual, a view that is challenged in this article. However, since there are no widely accepted alternative ways to describe the problems in question, I have used current terms.

## The Nature of Mental Health Problems

In contrast to the mainstream position, I and other critics suggest that mental health problems are not equivalent to general medical conditions (Valenstein, 1998; Szasz, 2000; Whitaker, 2002; Moncrieff, 2020). Although human beings are embodied creatures, and all human activity depends on biology, none of the situations we call mental disorders have been convincingly shown to arise from a biological disease, or, putting it another way, from a specific dysfunction of physiological or biochemical processes.

The abundance of research into the biological basis of mental disorders means it is difficult to challenge every new claim or theory, yet fundamental flaws have been identified in key areas of research. For example, genetic research with families and twins has overlooked important confounders and positive findings have been highlighted while negative ones have been buried (Rose et al., 1984; Joseph, 2003). More recent genome wide studies produce negligible evidence for any relevant genetic effects (Latham and Wilson, 2010; Moncrieff, 2014). The most consistent finding in biological psychiatry is that people diagnosed with schizophrenia have smaller brains and larger brain cavities than people without, and this has recently been shown to be due, at least in part, to the effects of antipsychotic treatment (Fusar-Poli et al., 2013). Any remaining differences are likely accounted for by intellectual ability and other uncontrolled factors (Moncrieff and Middleton, 2015). Biochemical research also fails to support widely held beliefs that mental disorders are caused by abnormalities of specific neurotransmitters (Valenstein, 1998). The hypothesis that depression is caused by serotonin deficiency is not supported by evidence from any of the principle areas of research into depression and the serotonin system (Moncrieff et al.). Evidence on dopamine also fails to confirm the dopamine hypothesis of schizophrenia or psychosis, though dopamine is known to be involved in arousal mechanisms that are likely to be awry in someone who is acutely psychotic (Moncrieff, 2009; Kendler and Schaffner, 2011).

Instead of viewing mental disorders as biological conditions that are inherent in individuals, I suggest we need to understand them as problems of communities or societies. If we do this, we will see from the following account of the evolution and functions of the mental health system, the principal problems we refer to as mental disorders consist, from a societal point of view, of dependency and disruptive behaviour. It is true that these problems can be caused by medical conditions. Occasionally, brain diseases, such as dementia and Huntingdon’s chorea produce behaviour that is aggressive or socially undesirable, and many physical diseases reduce people’s ability to maintain themselves. Indeed, for centuries, the institutions that developed to accommodate the mentally disturbed, also provided for people with neurological conditions, and sometimes still do (Rehling and Moncrieff, 2020). Moreover, in most countries, people with dementia, a neurological disease, are treated by psychiatrists rather than neurologists.

However, in the situations we routinely refer to as “mental disorders”, no disease can reliably be found. It is in the nature of human beings to react to their environment in different ways. Some people behave in ways that are bizarre, difficult to understand and sometimes troublesome for others, and some people are more productive and efficient than others. Rather than representing these problems as the manifestations of as yet undiscovered brain diseases, I suggest that “mental illness” is simply the collection of challenging situations that remain when those that are amenable to the criminal justice system and those that are caused by a specific, medical condition are taken out of the picture (Moncrieff, 2020).

In what follows I accept the view that many of our current mental troubles are consequences of the particular socio-economic conditions of late capitalism, and the way in which these consequences are construed (Davies, 2011; Cohen, 2016; Davies, 2017). However, in contrast to the purely social constructionist view, I also assume that some are perennial features of human life and occur across different sorts of societies with varying economic bases.

## Mental Health Problems From a Social and Economic Perspective

In line with this view, the mental health system can be viewed as a social response to the set of problems we refer to as “mental disorder” or “illness”. Some of these are problems for any modern society, whether capitalist, socialist or something else. Some are specific to capitalism. Though much debated, Marxist theory suggests that social institutions (the superstructure) reflect the need to support the prevailing economic system (the base) of each society and historical era (Harman, 1986). Therefore, institutional functions need to be understood in the context of the economic system in which they are embedded.

One of the functions of mental health services is to provide support and care for people when they are unable to look after themselves. Just like people with a severe physical disability, learning difficulties or neurological disease, people who have a serious mental disorder that would nowadays be referred to as schizophrenia, bipolar disorder or severe depression, are sometimes unable to wash or dress themselves, to manage money, shop, cook or maintain their environment in a habitable condition. The disability may be temporary, and many recover or improve, but for some it is long-term.

Serious mental disorder can also involve people behaving in ways that are disruptive or dangerous to the lives of others. Managing this behaviour to ensure social harmony is something societies have endeavoured to address long before the advent of capitalism, and is one of the principle functions of the mental health system. As legal scholar (and subsequently notorious lawyer), Alan Dershowitz, commented: “it is a fairly constant phenomenon in most societies that dangerous and bothersome people will be isolated by one means or another” (Dershowitz, 1974) (P 58). English history records how local, informal procedures aimed at managing dangerous and disruptive behaviour evolved to address lacunae in the criminal law, which included the difficulty of convicting people who were too confused, distracted or deluded to understand the justice system or respond to punishment. These informal procedures were gradually codified into formal law regarding the care and control of the “insane” (Dershowitz, 1974).

Disturbed and disruptive behaviour is not just a social nuisance, however, it potentially affects the processes of production that form the basis of modern societies. The individual who is acutely paranoid or severely depressed, for example, is unlikely to be able to work, or at least to work efficiently, and family members, too, may be prevented from working because of the disruption caused to their lives. Moreover, someone who is severely mentally disturbed may frighten and upset those around them, preventing people from feeling secure and motivated enough to satisfy the requirements of labour, and potentially jeopardising the whole system of modern production.

The more common, yet less visible social consequence of mental health problems that is specific to capitalist societies is not being able to support oneself financially. Capitalism depends on the majority of people earning their living through wage labour, and to be of use to capitalists, workers have to generate more wealth or value than they earn–what is known as “surplus value”. If an individual falls below a certain level of productivity, it is no longer worth the expense of employing them. However, people who are unable to participate in productive labour that generates “exchange value” may nevertheless be able to engage in other useful activities and create “use value”. They are not incapable of work, just incapable of doing the sort of work that is available in an advanced capitalist economy. Some of these people are part of the “industrial reserve army”, who are recruited into work at times of labour shortage, and who help capitalists to keep wages down to maximise profit, but others, whom Marx referred to as the “demoralised, the ragged”; are unable to perform capitalist work on any terms (Marx, 1990) (p. 797).

The inability to earn associated with mental health problems may be temporary, lasting for the few weeks or months that the episode of madness, depression or stress endures, or it may be longer-lasting. Even if it is temporary, it may be recurrent, and the occurrence and duration of episodes is highly individual and unpredictable, making it difficult for those without highly supportive employers to sustain employment. There is no mechanism integral to capitalism to provide for people who are not employed, but capitalist economies have developed systems of welfare through the course of the last century, including the provision of financial support to the those who are classified as medically sick or disabled (Matthews, 2018).

## The Mental Health System and the Welfare State

The mental health system, along with physical health services, education and the criminal justice system, fulfil certain social needs and thereby produce “use values” in the Marxist sense. If these services are provided capitalistically, that is by private firms that generate and accumulate capital through the extraction of surplus value, they also produce “exchange value”. In modern capitalist societies of all political hues, a large part of these services are funded and coordinated by the state, both because a significant section of the population cannot afford them, and because of the level of organisation required. They may be provided by state enterprises or by private firms or charitable organisations, and they are often referred to collectively as the Welfare State.

Marxist commentators on the Welfare State highlight how it contributes to the social reproduction of the capitalist system by ensuring that there is a supply of healthy, educated and disciplined workers (Gough, 1979; O'Connor, 1973). These activities indirectly facilitate productive labour and the process of capital accumulation. The welfare state also ensures social harmony, by providing for the old and sick and sustaining those who will never enter the workforce, for example. These expenses are what Marx referred to as the “*faux frais* [incidental expenses] of capitalist production” (Marx, 1990) (p. 797). They are not associated with capitalist production *per se*, but can be viewed as a means of legitimation of the system, since, by preventing people from dying on the streets, they ensure the continuation of capitalist relations of exploitation and domination through hegemony rather than force (Higgs, 1993). Other Marxists highlight how the welfare state resulted from class struggle, and represents a concession to the working class inspired by the threat of revolution (Ferguson et al., 2002; Matthews, 2018), and others have pointed out how many functions of the welfare state are necessary for social reproduction in any modern economic system, and are not specific to capitalism (Cowling, 1985).

Most welfare state spending is not directly productive as it is provided either by public enterprises, which do not generate surplus value, or, if provided by the private sector, capital accumulation is constrained by the limits of public funding and taxation. Welfare services embody a contradiction, therefore, and represent both a pre-requisite for the continued existence of capitalism, and, at the same time, a drain on the surplus; “both a condition of capital accumulation and a subtraction from it” (Pierson, 1996) (p. 581) (O'Connor, 1973). This has led some to argue that the welfare state potentially undermines capitalism in the long-run (Gough, 1979; Bennett et al., 2009).

The philosophy behind the creation of the welfare state in the mid 20th century, as espoused by Keynes and the social democratic regimes that took up his ideas, was that it was the duty of the state to intervene and alleviate problems such as poverty and unemployment. It was the state’s responsibility to ensure there were employment opportunities, education, housing and healthcare available to all (Higgs, 1993). During the 1970s, however, the welfare state came to be seen as contributing to or even causing the economic crisis of capitalism, and regimes all over the world started to bring in measures to reduce its costs. This commonly involved the privatisation of state services, since the private sector could employ people at lowers costs due to longer hours, worse pay and conditions. It also involved a reorientation of the philosophy behind the welfare state, which involved shifting responsibility from the government to the individual. Relatively generous and automatic unemployment or social security benefits were phased out, for example, and in their place individuals had to prove their entitlement, which involved demonstrating either a willingness to work, or an incapacity for work (Higgs, 1993).

Much of the mental health system predates the creation of the welfare state; indeed it prefigures other aspects of the welfare state in its role in producing a social environment conducive to the accumulation of capital. However, as a state-subsidised enterprise, it can usefully be considered as part of the welfare state, and as with other sectors, the provision of services for the seriously mentally ill has been increasingly transferred from the state to the private sector over recent decades.

## The functions of the mental health system

### Maintaining Order and Providing Care

The mental health system in England evolved out of the Poor Laws that were enacted from the Tudor period in order to manage the problems created by the expropriation of the agricultural population, which was the first step necessary to provide the labour needed for capitalism, as depicted by Marx in Das Capital (Marx, 1990). The Poor Laws provided material and financial assistance or “relief”, raised through local taxes, to families who could not provide for themselves, including in those instances in which a member of the family was mentally incapacitated. Poor Law officials also helped to keep the community safe and secure, and could use the money at their disposal to pay for the confinement of local people felt to be dangerous in various settings, such as a neighbouring household or, if necessary, a prison or prison-like establishment such as a “House of Correction” (Fessler, 1956; Rushton, 1988).

Public mental institutions, known as “asylums”, arose in the context of an austerity drive in the early 19th century. This was intended to reduce the welfare burden by ending the system of “outdoor relief” that supported people in their own homes, and making state support contingent on entering the forbidding and highly stigmatised Workhouse, a policy encapsulated in the 1834 Poor Law Amendment Act [although some local authorities continued to pay “outdoor relief” (Forsythe et al., 1996)]. With the rise of the Workhouse, the “deserving” poor, who could not work by dint of mental derangement or impairment among other causes, needed to be separated from the “undeserving” poor - those deemed capable of work. The former were diverted to the new system of public asylums for treatment and cure that were constructed all over England during the middle of the 19th century, while the latter were made to do hard labour in exchange for their upkeep in the Workhouse (Scull, 1993).

The system was publicly funded because the costs of care and confinement were way beyond the majority of families, and because, as historian, Andrew Scull, suggests, building on the work of Michel Foucault, it was part of the means of establishing a disciplined workforce that had the requisite motivation to be put to work as wage labourers in the service of Capital (Foucault, 1965; Scull, 1993). Asylums provided a secluded place where people whose behaviour was socially disruptive but not obviously criminal could be contained, but they also provided care and sustenance for those who were too confused, chaotic or apathetic to be put to work in the Workhouse or driven out to scrape a living together in the harsh world of Victorian England. Despite widespread myths to the contrary, people who were simply eccentric or socially deviant (e.g. unmarried mothers) were not routinely admitted to the public asylums unless their behaviour posed significant problems (Rehling and Moncrieff, 2020).

The need for the State to provide care and containment arose partly because the capitalist system of wage labour meant there was little spare capacity within the family or community to look after someone who could not look after themselves (Wright, 1997). All modern societies that rely on industrial production and a large workforce have similar requirements and allowing the disturbed and confused to roam the streets or rot away due to lack of care would quickly undermine the legitimacy of any system. Persuading people to work in a capitalist manner towards the enrichment of others arguably requires greater motivation and discipline, however, especially if, as was the case at the beginning of the capitalist era, people are not used to doing so. Early capitalism, therefore, produced a particular imperative for the management of the seriously mentally ill, which is manifested in the vast amount of public resources expended on the asylum system in the 19th century.

Although the roots of this system are political and social - “moral” according to Foucault - since the 19th century it has presented itself as a medical endeavour directed at medical problems. Foucault suggested that the medical framework was superimposed onto the system in order to give it the legitimacy associated with science. He referred to psychiatry as a “moral enterprise overlaid by the myths of positivism” (Foucault, 1965) (p. 276). In a modern liberal society where the rights of the individual are pre-eminent, psychiatry can only fulfil its functions by presenting itself as a technical activity that is immune to political considerations. The medical nature of psychiatric terminology and knowledge obscures the values and judgements that are embedded in its practical execution (Ingelby, 1981). It enables interventions that are designed to curb or control unwanted behaviour to be conceptualized as medical treatments intended to benefit the recipient rather than the people who are disturbed by the individual’s behaviour. It also extends the prerogative of the sick role, with its entitlement to care, to those who are unable to care for themselves, but where no obvious physical disease can account for their incapacity, and where the entitlement might, therefore, be questioned.

### Modern Developments

The large public asylums were scaled down and finally closed from the 1980s onwards, and the official story declares that this process of deinstitutionalisation, as it was known, demonstrates the efficacy of modern drug treatments and confirms the validity of the medical view of mental disorder (Cookson et al., 2005). A Marxist analysis, on the other hand, suggests that the institutions were closed because of the desire to reduce public spending (Scull, 1977). It is now apparent that although the new drugs may render some people more subdued, they rarely enable people to become fully independent. A study published in 2005, for example, found that in 1998, more people were dependent on state and private services due to mental health problems than in 1898 (Healy et al., 2005). Instead, long-term psychiatric patients are now placed in other institutions - smaller, privately-run but state funded residential and nursing homes, for example, as well as private psychiatric hospitals, secure units and prisons, and many rely on the care and support of family members or paid carers (Priebe et al., 2005). Many subsist on financial support from the state, the new version of “outdoor relief”.

Deinstitutionalisation was, therefore, partly an exercise in transferring provision for the long-term mentally disabled from the state to the private sector. The income still largely derives from the state, but the organisation of these services into private companies has enabled them to become a potential source of capital accumulation through the exploitation of employees.

### Welfare

The vast majority of people who are currently diagnosed with a mental disorder cause no trouble for other people and have no difficulty looking after themselves on a day-to-day basis but are not able to work and so rely on financial support provided through the state welfare system. Welfare payments have become an important part of the mental health system and illustrate how conceptualising certain problems as mental illness or disorder disguises the flaws of the capitalist system, thus helping to suppress resistance to it.

Marxist analysts of disability have pointed out how capitalism constructs disability or dependency as a social problem. In pre-capitalist societies, the distinction between the dependent and independent was not clear-cut. Most people could produce “use value”, contributing to the maintenance of the family and community in some fashion. In a capitalist society, in contrast, people are either fit to be exploited or they are unemployable (Finkelstein et al., 1981; Oliver, 1999; Slorach, 2011; Bengtsson, 2017). One of the major roles of the welfare state is the provision of financial or material support for those who cannot work intensively and productively enough to generate surplus value.

Sickness and disability payments were introduced in most western countries in the middle of the 20th century and have been rising rapidly since the 1980s, despite efforts to curb them (Kemp et al., 2006; Niemietz, 2016). Much of this rise is accounted for by the increase in people claiming benefits for mental health problems, particularly those classified as depression or anxiety (Waddell and Aylward, 2005; Kemp et al., 2006; Brown et al., 2009; Danziger et al., 2009). In the United Kingdom in 2008, it was estimated that the total cost of sickness and disability-related worklessness among the working age population was more than the cost of the whole of the National Health Service (Black, 2008). By 2014, almost half of United Kingdom claimants were classified as having a mental disorder as the reason for their claim, which was by far the largest category of causal medical conditions. Claims made due to a mental disorder doubled between 1995 and 2014, while claims made for most other types of medical conditions fell. These claims were predominantly long-term (Viola and Moncrieff, 2016). Similarly in the United States, claims for disability payments due to mental health problems have increased at a faster rate than claims for other medical conditions, and by 2005 they accounted for around a third of claims made to the major disability benefit schemes (Danziger et al., 2009). Again, once on disability benefits, people rarely go off them (Joffe-Walt, 2013).

The rise in disability payments to people with common mental disorders like anxiety and depression is paralleled by the phenomenal rise in antidepressant prescribing that has occurred since the early 1990s throughout the world. Consumption of antidepressants more than doubled in the United Kingdom between 1998 and 2010, for example (Ilyas and Moncrieff, 2012), having previously risen by more than three times from 1988 to 1998 (Middleton et al., 2001). There have been similar rises in many OECD countries (Organisation for Economic Development, 2020). Over the past few decades, an increasing proportion of people have been prescribed these drugs on a long-term basis (Mars et al., 2017; Taylor et al., 2019).

Studies of employment have also shown that receiving treatment for a mental health problem is associated with people taking more time off and being less likely to return to work than people who do not receive treatment (Dewa et al., 2003). It appears, therefore, that in many high income countries, including the United Kingdom and US, large numbers of people become economically inactive and are classified as being long-term mentally ill. They receive financial benefits and prescriptions for psychiatric drugs, and some may receive psychological therapy.

These recent trends illustrate the relationship between welfare and capital accumulation. During the period of Neoliberalism the ruling class has pushed back against the concessions that workers won during the mid 20th century in order to increase or maintain profit margins (Harvey, 2005; Glynn, 2006; Boltanski and Chiapello, 2018). This has been achieved by relocating many manual industries to countries where labour costs are cheaper, and by increasing the intensity or productivity of the work that remains (Office for National Statistics, 2018).

People have to work harder than they did in the past, their output and performance is constantly scrutinsed, and there is the constant threat of losing one’s jobaltogether, especially for the increasing number of people employed on a casual or “self-employed” basis. The work environment requires workers to be more and more robust, efficient and compliant (Dardot and Laval, 2017). This applies to the public sector too, which has been remodelled on the private sector since the 1990s (Ironside and Seifert, 2004). Whereas previously there may have been a niche for the less productive in state enterprises, such as the UK’s National Health Service (NHS), these now engage in intense performance monitoring and take a more disciplinarian approach to the workforce, resulting in a culture of “fear and blame” and a “demotivated workforce with low morale” (Stevenson and Moore, 2019) (p. 1). It is not surprising, therefore, that increasing numbers find they cannot tolerate the demands of work as it is currently organised.

Neoliberal capitalism increases the need or demand for disability benefits, therefore, but at the same time it attempts to restrain those benefits, which represent a drain on the overall surplus. In the United Kingdom, for example, the government has introduced more stringent criteria for qualifying as sick or disabled, abolished certain allowances, capped others, and set benefit rises below inflation (UNISON, 2013). Such measures are in constant tension with the fact that the alternative of working on the open market is less achievable for many, and hence attempts to restrain spending are barely successful (Office for Budget responsibility, 2019).

Capitalism creates redundant workers out of those people who can work, but are not productive enough to produce the desired amount of surplus value due to physical or mental disability (Finkelstein et al., 1981; Oliver and Flynn RJL, 1999). State-funded sickness and disability benefits disguise this structural unemployment–unemployment that is inherent to the current stage of capitalism (Beatty et al., 2000; Roberts and Taylor, 2019). In the US, this activity has become a new industry, with states paying businesses to help move people from state-funded social security to federally funded disability programmes (Joffe-Walt, 2013).

This process of exclusion from the productive workforce deprives people of a feeling of connection with and investment in their community, thus contributing to people becoming marginalised and demoralised, which is then labelled as mental illness. In this way, unemployment and low productivity are constructed as the fault of the individual (albeit a biological rather than a moral fault), rather than a systemic problem that reflects the prioritisation of profit over participation (Davies, 2017). The welfare system also solidifies people’s identity as “spoiled” or damaged; as being incapable. Like the asylums of the 19th century, it keeps the non-working population quiet and secluded so the rest can be effectively exploited.

### The Promotion of Hegemony

Underpinning the previously described functions of the mental health system is the idea that the situations concerned are medical conditions, with the implication that they originate in the body and thus absolve individuals of responsibility for their behaviour, and justify the forcible modification of that behaviour by others (Moncrieff, 2020). Although we have seen that this position is not supported by scientific evidence, it is widely embraced and its acceptance helps to legitimise the social and political status quo.

Construing life difficulties as an illness in what Nikolas Rose has called “the psychiatric re-shaping of discontent” (Rose, 2006) (p. 479) has long been recognised as a political strategy that silences protest and inhibits change. This was pointed out in the 1960s and 1970s by social scientists who explored the creeping medicalisation of society (Zola, 1972; Illich, 1976; Conrad and Schneider, 1980), along with “antipsychiatry” thinkers (Laing, 1967) and has been explored more recently by critics of neoliberalism (Fisher, 2009; Cohen, 2016; Davies, 2017). This strategy has been employed in socialist as well as capitalist countries. As William Davies points out, unhappiness has “political and sociological qualities that lend it critical potential” (Davies, 2011). To construe it as an illness, to label it as “clinical depression” as it is in neoliberal, western societies, as anxiety as it was for much of the 20th century (Healy, 2004), or neurasthaenia as it was in the Soviet bloc and communist China (Kleinman, 1982; Skultans, 2003), is to declare that it is not reasonable, to see it as something to be eradicated, rather than understood. Viewing worry, distress and misery as a medical condition isolates the individual as a patient who needs to be cured of their internal flaws. It cuts them off from understanding the social implications of their feelings, and it prevents society from understanding epidemics of mental health problems as “commentaries on social life” (Davies, 2017) (P 205).

As already noted, there has been a huge expansion in the numbers of people receiving mental health diagnoses and treatments in high income countries over recent decades with dramatic increases in the use of antidepressants, in particular, but also of stimulants (commonly prescribed for a diagnosis of ADHD), new anti-anxiety agents and drugs usually associated with the treatment of more severe disorders, such as antipsychotics (Ilyas and Moncrieff, 2012). Seventeen per cent of the population of England are now prescribed an antidepressant alone (Taylor et al., 2019).

There are some obvious drivers of this trend, such as the pharmaceutical industry, whose marketing activities have been facilitated both by the arrival of the Internet, and by political deregulation, including the repeal of the prohibition on advertising to consumers in the US and some other countries in the 1990s (Davies, 2017). Despite the fact that there is no evidence of an imbalance or abnormality of brain chemicals or any other biological abnormality in people with depression (Kennis et al., 2020; Moncrieff et al., 2021), the industry, aided and abetted by professional organisations such as the American Psychiatric Organisation and the UK’s Royal College of Psychiatrists (APA, 2018; Royal College of Psychiat, 2009), has succeeded in persuading the general public that unhappiness and discontent arise from a faulty brain. Surveys conducted in the US and Australia in the 2000s, for example, showed that 85 and 88% of respondents respectively endorsed the idea that depression is caused by a chemical imbalance (France et al., 2007; Pilkington et al., 2013).

Political institutions have also embraced the idea that human reactions to difficult circumstances can be understood as mental health problems. The United Kingdom government’s initiative on “transforming children and young people’s mental health” for example (NHS, 2021), is premised on the idea that the source of stress, anxiety and behaviour problems among the young is not the conditions they grow up in or the highly competitive nature of the modern educational system, but individual flaws or weaknesses that can be addressed through treatment designed to help the individual to adjust and assimilate. Mental health support teams have been introduced into schools to “provide early intervention on some mental health and emotional wellbeing issues, such as mild to moderate anxiety” and referrals to NHS services for more severe problems. Inevitably, this will lead to increasing numbers of pupils being given a potentially stigmatising diagnostic label and pharmaceutical treatments, which are unlikely to have net benefits for most of them but certainly have risks and dangers (Kazda et al., 2021).

Capitalism requires a certain level of dissatisfaction in order to operate smoothly and maintain consumption. People need to be persuaded that their lives are lacking in some way, and neoliberalism, with its rolling back of state responsibilities, has exaggerated this tendency (Davies, 2011). The “privatisation of public troubles and the requirement to make competitive choices at every turn” (Hall et al., 2013) (p. 12) breed perpetual feelings of insecurity and inadequacy that establish the demand necessary to stoke capital accumulation. The construction of the ideal neoliberal subject as an informed and intelligent consumer, who is fully responsible for their own wellbeing, both creates the conditions for increasing personal stress, in what has been called a “malady of responsibility” (Dardot and Laval, 2017) (P 292), and encourages people to look for solutions in the consumption of pharmaceuticals and other easily marketable products, such as short-term therapy (Davies, 2017).

Competition, the basis of the capitalist system, creates winners and losers. Fear of failure is therefore a constant source of anxiety for the modern individual, and failure itself so often the precipitant of the demoralisation and hopelessness that is called depression (Ehrenberg, 2010; Dardot and Laval, 2017). “Depression is the shadow side of entrepreneurial culture,” said Marxist author Mark Fisher, “what happens when magical voluntarism confronts limited opportunities” (Fisher, 2012).

Presenting this situation as individual deficiencies rather than a systemic by-product helps obscure its political and economic origins. The language of mental health and mental illness or disorder can be thought of, therefore, as an “ideology”, in the Marxist sense that these concepts help to obscure real underlying tensions and conflicts, and render the population amenable to viewing them as relatively simple, technical problems that should be left to experts. As Bruce Cohen points out, “biomedical ideology has become the dominant “solution’’ to what are social and economic conditions of late capitalism’’ (Cohen, 2016) (p. 91). Authors who have described this phenomenon as “psychiatrization” highlight how it leads to numerous personal and social consequences from the creation of individual dependency to the diversion of needed resources from other areas of health and social services (Beeker et al., 2021), but most importantly, from the Marxist point of view, it disguises “failed policies” (Conrad, 1992) (p. 7).

The current “mental health movement”, with its encouragement to conceive of our understandable reactions to an increasing array of social problems, including unemployment, school failure, child abuse, domestic violence and loneliness as individual pathology requiring expert, professional treatment, promotes an ideology that helps legitimise existing social and economic relations by diverting attention from the problems themselves. In this way, it acts as a hegemonic tool for the capitalist system that now dominates most of the globe. It has been successful in moulding public attitudes and gaining political support, despite efforts of some mental health campaigners, professionals and academics to expose its political implications and to present other ways of understanding the difficulties we currently refer to as mental disorders (Johnstone et al., 2018; Guy et al., 2019).

## Social Responses to Mental Health Problems

As this analysis illustrates, how society responds to the problems posed by dependency and troublesome behaviour is potentially contentious. For Foucault, and medical sociologists, such as Peter Conrad, one of the important consequences of the medicalisation of such problems is to render them morally and politically neutral (Foucault, 1965; Conrad, 1992). The concept of mental illness provides a justification for using force against people whose behaviour is antisocial or dangerous, but who are too confused or irrational to be appropriate for the criminal justice system. It also authorises support for people who do not qualify for care or welfare by virtue of being old or physically sick or disabled. Presenting these responses as medical activities that are the rightful and exclusive terrain of qualified, medical specialists shields them from being questioned or challenged. As the psychiatrist and critic, Thomas Szasz pointed out, the psychiatric system performs its functions “in a manner that pleases and pacifies the consciences of politicians, professionals and the majority of the people” (Szasz, 1994) (p. 200). It also has a wider hegemonic role in the maintenance of capitalism, along with other socio-economic systems, by locating the sources of individuals’ unhappiness and discontent within their own brains, rather than in their external circumstances, individualising “what might otherwise be seen as collective social problems” and thereby letting the political and economic system off the hook (Conrad, 1992) (p. 224).

On the other hand, some left-wing analysts, notably Peter Sedgewick, point out that this position enables capitalist governments to cut disability benefits and reduce other resources available for people affected by mental health problems (Sedgewick, 1982). While this may be theoretically possible, it depends on Sedgewick accepting the view that mental disorders are essentially equivalent to neurological diseases.

Apart from the lack of evidence that this is the case, it is difficult to accept that all dependency and disruptive behaviour is caused by a physical disease. If it is not, (Moncrieff, 2020)? then surely we need a more transparent system of control and care, that acknowledges the ethical and political dilemmas involved and is based on widespread democratic debate informed particularly by the voice of the system’s recipients. Such a system would have to balance the need to restrict people’s behaviour when it becomes a nuisance or danger to other people, with the individual’s legitimate interests to live in the way they want to live (19). We also need an alternative to the sick role in order to fairly and transparently distribute resources and care to people who are unable to be financially or practically independent, without having to deem them as being biologically flawed (Cresswell and Spandler, 2009).

## Reflections on Capitalism

This analysis suggests that the mental health system can be understood as part of a wider system of social reproduction through which modern societies produce a fit, capable and amenable workforce and ensure social harmony. The particular means of social reproduction depend on the economic and social form that each society takes. Some aspects of the mental health system are an enduring response to perennial social problems that cut across different epochs, political systems and cultures. These have not been fundamentally changed by the introduction of modern medical perspectives and interventions. For hundreds of years, English Poor Law officials grappled with how to help a family whose breadwinner had become mentally incapable, or how to protect the community from someone who was behaving irrationally and unpredictably (Rushton, 1988). Supporting the chronically dependent and controlling chaotic and disruptive behaviour remain the main functions of the modern mental health system.

On the other hand, some trends are distinctive of capitalism in general and neoliberal capitalism in particular. The modern welfare state emerged, in part, to compensate those who cannot work intensively and productively enough to earn a living through wage labour. The concept of mental illness enables a system that is justified by the nature of physical sickness and disability to incorporate people who are disorganised, demoralised, slow, antisocial, chaotic or unmotivated–factors whose significance clearly varies according to the nature of the work that is available. Some of these people may be recruited into the work force during an economic boom, and in the mid 20th century, when conditions for labour were more favourable, even those people diagnosed with severe mental conditions such as ‘schizophrenia’ had a reasonable chance of employment (Warner, 2004).

During the decades of neoliberal capitalism, however, as labour entitlements have been rolled back and work has become more competitive and exploitative, increasing numbers of people have become economically inactive for long periods. It is patently absurd to imagine that the quarter of the population who have been diagnosed with a mental illness (Health and Social Care Information Centre, 2015), or the fifth who take antidepressants (Taylor et al., 2019) have an as yet unidentified brain disease. Instead, this situation reflects the changing structure of contemporary capitalism. Disability support disguises the way in which capitalism narrows the opportunities for people to contribute to the productive efforts of society, thereby relegating large numbers of people into a surplus population that has no investment in its own community. The transformation of post-industrial populations into mental patients represents the economic and social marginalisation of a large segment of society. Rejecting the medicalisation of so-called mental health problems is a necessary step in revealing some of the fundamental contradictions of capitalism and laying the groundwork for political change.
